# Population genomics of the eastern cottonwood (*Populus deltoides*)

**DOI:** 10.1002/ece3.3466

**Published:** 2017-10-10

**Authors:** Annette M. Fahrenkrog, Leandro G. Neves, Márcio F. R. Resende, Christopher Dervinis, Ruth Davenport, W. Brad Barbazuk, Matias Kirst

**Affiliations:** ^1^ School of Forest Resources and Conservation University of Florida Gainesville FL USA; ^2^ Plant Molecular and Cellular Biology Graduate Program University of Florida Gainesville FL USA; ^3^ Horticultural Sciences Department University of Florida Gainesville FL USA; ^4^ Biology Department University of Florida Gainesville FL USA; ^5^ University of Florida Genetics Institute University of Florida Gainesville FL USA; ^6^ Present address: RAPiD Genomics LLC 756 2nd Avenue Gainesville FL 32601 USA

**Keywords:** eastern cottonwood, exome capture, genetic diversity, local adaptation, population structure, *Populus deltoides*

## Abstract

Despite its economic importance as a bioenergy crop and key role in riparian ecosystems, little is known about genetic diversity and adaptation of the eastern cottonwood (*Populus deltoides*). Here, we report the first population genomics study for this species, conducted on a sample of 425 unrelated individuals collected in 13 states of the southeastern United States. The trees were genotyped by targeted resequencing of 18,153 genes and 23,835 intergenic regions, followed by the identification of single nucleotide polymorphisms (SNPs). This natural *P. deltoides* population showed low levels of subpopulation differentiation (*F*_ST_ = 0.022–0.106), high genetic diversity (θ_W_ = 0.00100, π = 0.00170), a large effective population size (*N*_e_ ≈ 32,900), and low to moderate levels of linkage disequilibrium. Additionally, genomewide scans for selection (Tajima's *D*), subpopulation differentiation (*X*^*T*^*X*), and environmental association analyses with eleven climate variables carried out with two different methods (LFMM and BAYENV2) identified genes putatively involved in local adaptation. Interestingly, many of these genes were also identified as adaptation candidates in another poplar species, *Populus trichocarpa*, indicating possible convergent evolution. This study constitutes the first assessment of genetic diversity and local adaptation in *P. deltoides* throughout the southern part of its range, information we expect to be of use to guide management and breeding strategies for this species in future, especially in the face of climate change.

## 1 INTRODUCTION

Forests are one of the main carbon sinks on Earth and have a critical role in the mitigation of climate change (Bellassen & Luyssaert, 2014). To fulfill this role, adequate management and conservation strategies are essential. Developing these strategies is challenging, because tree populations may not be able to adapt fast enough to environmental changes due to their long generation times (Aitken, Yeaman, Holliday, Wang, & Curtis‐McLane, 2008). Thus, in‐depth knowledge of genetic diversity and a better understanding of the genetic regulation of adaptation in forest tree species are essential to predict their fate (Sork et al., 2013). Uncovering genes and polymorphisms that determine adaptation can also support the development of genetically improved germplasm that is suitable to be used in forest plantations in new or in changing environments.

In the last decade, poplar trees have emerged as models for molecular studies in woody perennial plants (Taylor, 2002). The *Populus trichocarpa* genome, the first tree to be sequenced (Tuskan et al., 2006), created numerous opportunities for pioneering genomic studies in tree species. Several genomewide association, transcriptome, and population genomics studies have been reported for *P. trichocarpa*, contributing to the current knowledge about the species regulation of complex traits (McKown, Klápště, et al., 2014; Porth et al., 2013a, 2013b), extent of linkage disequilibrium (LD) and effective population size (Slavov et al., 2012; Zhou, Bawa, & Holliday, 2014), population structure (Geraldes et al., 2014; Slavov et al., 2012; Zhou et al., 2014), genetic diversity (Evans et al., 2014; Zhou et al., 2014), demographic history (Zhou et al., 2014), adaptation (Evans et al., 2014; Geraldes et al., 2014; Holliday, Zhou, Bawa, Zhang, & Oubida, 2016; Porth et al., 2015; Zhou et al., 2014), and sex determination (Geraldes et al., 2015). Studies in other poplar species (Stölting et al., 2013, 2015; Wang, Street, Scofield, & Ingvarsson, 2016a, 2016b) have significantly lagged behind. In fact, very limited population genetic information is available for widely distributed and ecologically and economically critical species such as *Populus deltoides*.

Species in the genus *Populus* are typically long‐lived with extensive geographic distribution and high gene flow through long‐distance pollen and seed dispersal (Ingvarsson, 2010). In existing studies, high levels of genetic diversity and limited population structure have been observed. Genetic structure is mainly driven by interspecific hybridization, isolation by distance, and natural selection (Evans et al., 2014; Geraldes et al., 2014; Slavov et al., 2012; Stölting et al., 2015; Wang et al., 2016b; Zhou et al., 2014). Adaptation has been shown to have a complex genetic architecture (Evans et al., 2014; Holliday et al., 2016; McKown, Guy, et al., 2014; McKown, Klápště, et al., 2014; Porth et al., 2015) and to be a driver of divergent evolution between species (Wang et al., 2016b). Contradictory estimates of LD decay have been reported for *P. trichocarpa*, with an early study on a reduced number of genes reporting fast LD decay below a threshold of 0.2 (200 bp, Wegrzyn et al., 2010). However, more recent genomewide studies reported an LD decay that extends to several kilobase pairs (3.0–7.5 kb; Slavov et al., 2012; Zhou et al., 2014).

Conducting population genomics studies of other poplar species provides the opportunity to further characterize the genus’ diversity, to discover novel genes involved in adaptation to environments not surveyed before, and to confirm previously identified candidate genes. Among poplars, *P. deltoides* (eastern cottonwood) is one of most ecologically important species for riparian ecosystems throughout its native range, spanning from the southeastern United States to southern Canada. This tree is also very important economically because it is one of the main species used in poplar breeding for the development of improved feedstocks for various industrial processes, including biofuel production (Stanton, Neale, & Li, 2010). Here, we report the first population genomics study conducted for this species, providing insight into the genetic diversity, LD and signatures of selection, and adaptation in *P. deltoides*. This study also provides a first glance at the species’ adaptation to the environment. This information will be useful to predict the future of this tree in its natural habitat and for selection of germplasm better adapted to overcome the challenges posed by a changing climate.

## 2 MATERIALS AND METHODS

### 2.1 Plant material and targeted sequencing

The *P. deltoides* Bartr. ex Marsh. (eastern cottonwood) population used in this study is composed of 579 individuals sampled in 15 states in central, southern, and eastern United States and maintained at the University of Florida. This population was genotyped using a sequence capture/next‐generation sequencing approach described in detail elsewhere (Fahrenkrog et al., 2017). Briefly, genomic DNA was extracted from leaf tissue using the DNeasy Plant Mini Kit (Qiagen, Valencia, CA, USA), and barcoded libraries were prepared for each sample. Pools containing 12 or 13 samples were prepared, and a set of 227,943 probes was used to capture 18,153 genes (with 204,180 probes) and 23,835 intergenic regions (with 23,835 probes). Pooled sequence capture was performed using the SureSelect Target Enrichment kit (Agilent Technologies, Santa Clara, CA, USA), following the manufacturer's protocol. The captured DNA was sequenced with the Illumina HiSeq 2000 Sequencing System (Illumina, San Diego, CA, USA). Sequencing reads were split by barcode, filtered, and trimmed by quality and aligned to a hybrid *P. trichocarpa*/*P. deltoides* reference genome using MOSAIK 2.2 (Lee et al., 2014).

### 2.2 Single nucleotide polymorphism identification

As described in detail in Fahrenkrog et al. (2017), single nucleotide polymorphisms (SNPs) were identified in the nuclear genome using three different variant callers: SAMTOOLS 1.1 (Li, 2011), FREEBAYES 0.9.15 (Garrison & Marth, 2012), and GATK 3.1 (DePristo et al., 2011; McKenna et al., 2010; Van der Auwera et al., 2013). A SNP set referred to as “consensus SNPs” hereafter was obtained from the overlap between callers, removing SNPs with a quality score below 50 and SNPs with mapping quality below 30. Genotypes with a quality score below 20 and depth below eight were set to missing. These consensus SNPs were used previously (Fahrenkrog et al., 2017) to identify a core population of 425 unrelated individuals and to assess population structure using STRUCTURE (Pritchard, Stephens, & Donnelly, 2000) and principal component analysis (PCA). Here, they were used to conduct an environmental association analysis (EAA, workflow overview available in Fig. S1). A subset of consensus SNPs was annotated with SNPEFF (Cingolani et al., 2012) for their predicted effect (Fahrenkrog et al., 2017).

The use of the consensus SNP set generated by the overlap of three variant callers was adopted to reduce the occurrence of false positives, but also resulted in the exclusion of many low‐frequency variants, which may bias estimates of population genetic parameters. Thus, a frequency‐unbiased SNP set was developed for the core population based on those loci identified by the GATK only. GATK SNPs with a quality score below 50; mapping quality below 30; strand bias (*p*‐value ≤ .00001); and end‐distance bias (*p*‐value ≤ .00001) were removed. Also, genotypes with a quality score below 20 and depth below eight were set to missing, and SNPs with more than 25% missing data were excluded. Filters were chosen following recommendations by Carson et al. (2014). This SNP set, referred to as “filtered SNPs” hereafter, was used to estimate population genetics parameters.

### 2.3 Linkage disequilibrium

Pairwise LD (*r* ^2^) between nuclear markers was calculated with PLINK 1.9 (Purcell et al., 2007) for each gene that contained two or more filtered SNPs in the population. LD decay with physical distance within genes was estimated based on sample size (*n*) and the parameter *C*, where *C* is the product of the population recombination parameter (ρ = 4*N*_e_ *r*) and the distance in base pairs (Marroni et al., 2011; Remington et al., 2001).

$$E\;\left(r^{2}\right)=\left[\frac{10+C}{\left(2+C)(11+C\right)}\right]\left[1+\frac{\left(3+C\right)\left(12+12C+C^{2}\right)}{n(2+C)(11+C)}\right]$$

Parameter *C* was estimated from the data using the nonlinear least squares (nls) function in R (R Core Team, 2015). LD decay with distance was estimated in the total population and for each subpopulation individually.

### 2.4 Genetic diversity, population differentiation, and signatures of selection

Individuals were assigned to subpopulations based on the ancestry coefficients previously obtained with STRUCTURE for the core *P. deltoides* population (Fahrenkrog et al., 2017). Samples with an ancestry coefficient above 80% for a specific subpopulation were assigned to that group, whereas the remaining individuals were labeled as admixed. Expected heterozygosity (*H*_E_), observed heterozygosity (*H*_O_), and pairwise population differentiation among subpopulations (*F*_ST_) were obtained with the 4P software (Benazzo, Panziera, & Bertorelle, 2015) as locus‐by‐locus and population mean estimates. Nucleotide diversity π, Watterson's estimator of nucleotide diversity θ_W_ (Watterson, 1975), and Tajima's *D* (Tajima, 1989) and Wall's *B* (Wall, 1999) statistics for detection of signatures of selection were calculated by gene with the PopGenome package for R (Pfeifer, Wittelsbürger, Ramos‐Onsins, & Lercher, 2014). Diversity (θ_W_) was also estimated for intergenic regions. All 4P and PopGenome analyses were conducted for the entire population as well as separately by subpopulation using the 555,673 filtered SNPs.

The measure of subpopulation differentiation *X* ^*T*^ *X* was calculated by locus using the BAYENV2 software (Günther & Coop, 2013) for the two subpopulations identified as the first level of hierarchical clustering in the STRUCTURE analysis (East‐K2 and West‐K2 subpopulations). BAYENV2 uses a variance–covariance matrix of allele frequencies among subpopulations (Ω) to correct for evolutionary history. Estimation of Ω with neutral SNPs is referred to as “neutral parametrization” (Lotterhos & Whitlock, 2014). For this purpose, a set of 1,800 intergenic (putatively neutral) loci was selected from the intergenic SNPs used to assess nuclear population structure. The 1,800 selected loci included only variants at least 15 kb apart, with minor allele frequency (MAF) >0.01 and call rate >80%. The Ω matrix was obtained by averaging the last matrix generated by five independent runs of 500,000 Monte Carlo Markov Chain (MCMC) cycles in the BAYENV2 program. The population differentiation statistic *X* ^*T*^ *X* was computed for the intergenic SNPs to obtain the *X* ^*T*^ *X* distribution for neutral loci. Additionally, *X* ^*T*^ *X* was estimated for 223,643 genic SNPs selected from the filtered SNPs after removing variants with MAF < 0.01, as recommended in the BAYENV2 manual (https://bitbucket.org/tguenther/bayenv2_public), and keeping only SNPs in the main 19 scaffolds of the genome corresponding to the 19 poplar chromosomes. *X* ^*T*^ *X* outlier loci were identified from empirical *p*‐values obtained based on the null *X* ^*T*^ *X* distribution provided by the intergenic SNPs.

### 2.5 Environmental association analysis

Association between markers and bioclimatic variables was analyzed in 168 unrelated individuals from the core population with known geographic coordinates for their sampling location (Table S1). Climate data for current conditions (~1950–2000) were obtained from the WORLDCLIM database (http://www.worldclim.org, accessed in January 2016) at 2.5 arc‐minutes resolution. These data included minimum, maximum and mean monthly temperature, monthly precipitation, altitude, and 19 bioclimatic variables, derived from monthly temperature and precipitation. Due to high correlation between environmental variables, a PCA was conducted with the R package FactoMineR (Lê, Josse, & Husson, 2008) to select one or two environmental variables with the highest contribution to the first four principal components (PCs) obtained separately for temperature and precipitation variables. The EAA was performed with two programs, LFMM (Frichot, Schoville, Bouchard, & François, 2013) and BAYENV2 (Günther & Coop, 2013), for comparison.

The LFMM program tests for correlation between environmental and genetic variation and corrects for the effect of population structure (latent factors) simultaneously. The number of latent factors to be estimated (*K*) needs to be defined by the user. To define the number of latent factors to be used in this study, different values of *K* (*K* = 2, 3, 4, 6, 8, 10, 12, 14, 16, 18, 20) were tested with two environmental variables. The association results obtained were very similar between all *K* values; thus, *K* = 2 was chosen for the analysis of all remaining environmental variables. Among the consensus SNPs segregating in the 168 individuals, a subset of 114,261 common SNPs (MAF > 0.05) was selected for this analysis after pruning by LD using an *r* ^2^ threshold of 0.8 and keeping only the SNPs in the main 19 scaffolds. The EAA was performed five independent times for each environmental variable for a total of 10,000 MCMC cycles, with 5,000 of these cycles corresponding to the burn‐in period. As described in the LFMM manual (http://membres-timc.imag.fr/Olivier.Francois/lfmm/files/note.pdf), *z*‐scores from the five runs were combined and a *p*‐value for the association between each marker and the environmental variable tested was obtained.

The BAYENV2 program tests for correlation between population allele frequencies and standardized environmental variables. To be able to perform this analysis, the 168 samples with known sampling location were grouped into 50 smaller populations based on similar latitude, longitude, and altitude, following the strategy used by Geraldes et al. (2014) (Table S1). The variance–covariance matrix, Ω, was estimated using 3,667 genic LD pruned SNPs (*r* ^2^ ≤ 0.2) with no missing data. Variants previously identified to be *X* ^*T*^ *X* outliers or significantly associated with an environmental variable after the analysis conducted with LFMM where not included in the SNP set used for the estimation of Ω. As before, the Ω matrix was obtained by averaging the last matrix generated by five independent runs of 500,000 MCMC cycles in the BAYENV2 program. To identify SNPs associated with environmental variables, BAYENV2 used 500,000 MCMC cycles to estimate a Bayes factor and the nonparametric Spearman's rank correlation coefficient ρ individually for each SNP—variable combination in five independent runs. This analysis was performed on a set of 72,969 SNPs with less than 25% missing data selected from the 114,261 SNPs also tested with LFMM. Bayes factor, and ρ estimates for every SNP were obtained from the median of the five independent runs. Markers ranked among the 1% highest Bayes factor and also among the highest 1% absolute value of ρ where considered to be strong candidates for adaptation to a given environmental variable.

### 2.6 Functional enrichment test

The sets of genes identified as possible selection candidates with different approaches (Tajima's *D* and *X* ^*T*^ *X* outliers, and environmental associations identified with LFMM and BAYENV2) were tested for gene function enrichment using Fisher's exact test implemented in the Populus Genome Integrative Explorer (PopGenIE; http://popgenie.org/; Sjödin, Street, Sandberg, Gustafsson, & Jansson, 2009). This database allows testing for enrichment for the gene ontology (GO) categories biological processes, molecular function, and cellular component, as well as for enrichment for protein families (PFAM), Kyoto Encyclopedia of Genes and Genomes (KEGG) annotations, and microRNA annotations. It also includes a test for enrichment for genes targeted by homeodomain transcription factors involved in secondary growth and wood development identified in a chromatin immunoprecipitation sequencing (Chip‐seq) study (Liu et al., 2015). A false discovery rate (FDR) correction for multiple testing was applied at a significance level of 0.05.

## 3 RESULTS

### 3.1 Pairwise population differentiation is low in *Populus deltoides*

Previously, we assessed nuclear population structure in a sample of 425 unrelated *P. deltoides* individuals, using 8,664 intergenic SNPs (Fahrenkrog et al., 2017). An ancestry coefficient cutoff of 0.8 was used to assign individuals to subpopulations, with samples with ancestry coefficients below this threshold being classified as admixed. Based on this and on the sampling location known for a subset of the individuals (168 of 425 samples), four geographically distinct subpopulations were identified (fig. 1 in Fahrenkrog et al., 2017; Table S2): (1) West: 43 samples, with 31 of them known to come from the States of Texas (TX), Oklahoma (OK), and Arkansas (AR); (2) West‐MR: 101 samples, with 82 from TX, AR, Louisiana (LA), Tennessee (TN), Mississippi (MS), Alabama (AL), and Georgia (GA), including most of the trees growing along the Mississippi River; (3) Center: 26 samples, with 10 from Northern Florida (FL); and (4) East: 177 samples, with 11 from South Carolina (SC) and North Carolina (NC). A group of 78 samples was considered admixed, including samples with different degrees of genome composition from the four subpopulations described above.

Pairwise population estimation of the fixation index (*F*_ST_) showed low differentiation between the two western subpopulations (*F*_ST_ = 0.022 for West vs. West‐MR) and higher differentiation between the remaining subpopulations (pairwise *F*_ST_ values = 0.097 for West vs. East; 0.106 for West vs. Center; 0.070 for West‐MR vs. East; 0.080 for West‐MR vs. Center; and 0.091 for East vs. Center) (Table S3). Differences among populations account for not more than 10% of the genetic variation in the total population.

### 3.2 Genetic diversity and effective population size in *Populus deltoides*

Natural poplar populations are expected to show high levels of genetic diversity because of their wide distribution, extensive gene flow, and low population differentiation (Ingvarsson, 2010; Ingvarsson, Hvidsten, & Street, 2016). Genetic diversity was measured in the *P. deltoides* population as a whole, and the four subpopulations described above separately. This analysis was performed using 555,673 SNPs obtained after filtering variants identified with GATK. Analysis with the 4P software (Benazzo et al., 2015) revealed expected heterozygosity (*H*_E_) values of 0.077 for the entire population, and 0.079, 0.077, 0.066, and 0.069 for subpopulations West, West‐MR, Center, and East, respectively. Observed heterozygosity (*H*_O_) was lower than *H*_E_ for the entire population, slightly higher for subpopulations West (*H*_O_ = 0.080) and West‐MR (*H*_O_ = 0.079), and similar for subpopulations Center (*H*_O_ = 0.066) and East (*H*_O_ = 0.069) (Table S4).

Two estimators of genetic diversity, Watterson's estimator θ_W_ (Watterson, 1975) and nucleotide diversity π (Nei & Li, 1979), were obtained for each gene with PopGenome (Pfeifer et al., 2014), using the 555,673 filtered SNPs. Diversity for all 17,633 genes analyzed was higher in the complete population (θ_W_ = 0.00100, π = 0.00170, number of segregating sites [*S*] = 32) than in any of the subpopulations (Table S4). Among the four subpopulations identified, highest and lowest diversity was found in subpopulations West‐MR (θ_W_ = 0.00095, π = 0.00099, *S* = 21) and Center (θ_W_ = 0.00052, π = 0.00082, *S* = 8), respectively. The Center subpopulation is also the group with the smallest sample size (*N* = 26), and therefore, allele frequencies calculated for this subpopulation might be inaccurate due to sampling error, affecting genetic diversity estimates. The mean diversity values obtained here are lower than diversity reported previously for three nuclear genes in *P. deltoides*, where θ_W_ ranged from 0.00160 to 0.00245 (Breen, Glenn, Yeager, & Olson, 2009). Nonetheless, these modestly higher diversity values reported previously are within the diversity range (0.00000 ≤ θ_W_ ≤ 0.00910) detected for genes in this study. Similarly, the mean θ_W_ of 0.0010 obtained for *P. deltoides* is lower than the mean θ_W_ of 0.0029 reported previously for genes in *P. trichocarpa* (Zhou et al., 2014), but of a similar magnitude. Diversity (θ_W_) was five to nine times higher when estimated using SNPs in 4,964 intergenic regions across the genome, with highest and lowest diversity featured by subpopulations West‐MR (θ_W_ = 0.0053) and East (θ_W_ = 0.0040), respectively.

Another goal of this study was to estimate the effective size of the population, which was expected to be large based on the extensive native range and long‐distance pollen and seed dispersal for this species (Slavov & Zhelev, 2010). In a diploid organism, the effective population size can be estimated based on the genetic diversity (θ_W_) and the per generation mutation rate (μ; Hartl & Clark, 2007). A per generation mutation rate of 3.75 × 10^−8^ was obtained by multiplying the estimated per year mutation rate of 2.5 × 10^−9^ (Ingvarsson, 2010) by a generation time of 15 years (Zhou et al., 2014). Because of its dependency on genetic diversity levels, effective population size was higher in the complete population (*N*_e_ ≈ 6,700), followed by subpopulations West‐MR (*N*_e_ ≈ 6,300), West (*N*_e_ ≈ 5,500), East (*N*_e_ ≈ 4,300), and Center (*N*_e_ ≈ 3,400) (Table S4). These estimates are of similar magnitude to *N*_e_ reported for three subpopulations in *P. trichocarpa*, ranging between 4,800 and 7,500 (Zhou et al., 2014). When using intergenic genetic diversity to estimate *N*_e_, the values ranged between ≈26,800 (East) and 35,100 (West‐MR; Table S4).

### 3.3 Genomewide patterns of nucleotide diversity, signatures of selection, and LD decay in *Populus deltoides*

**Figure 1:**
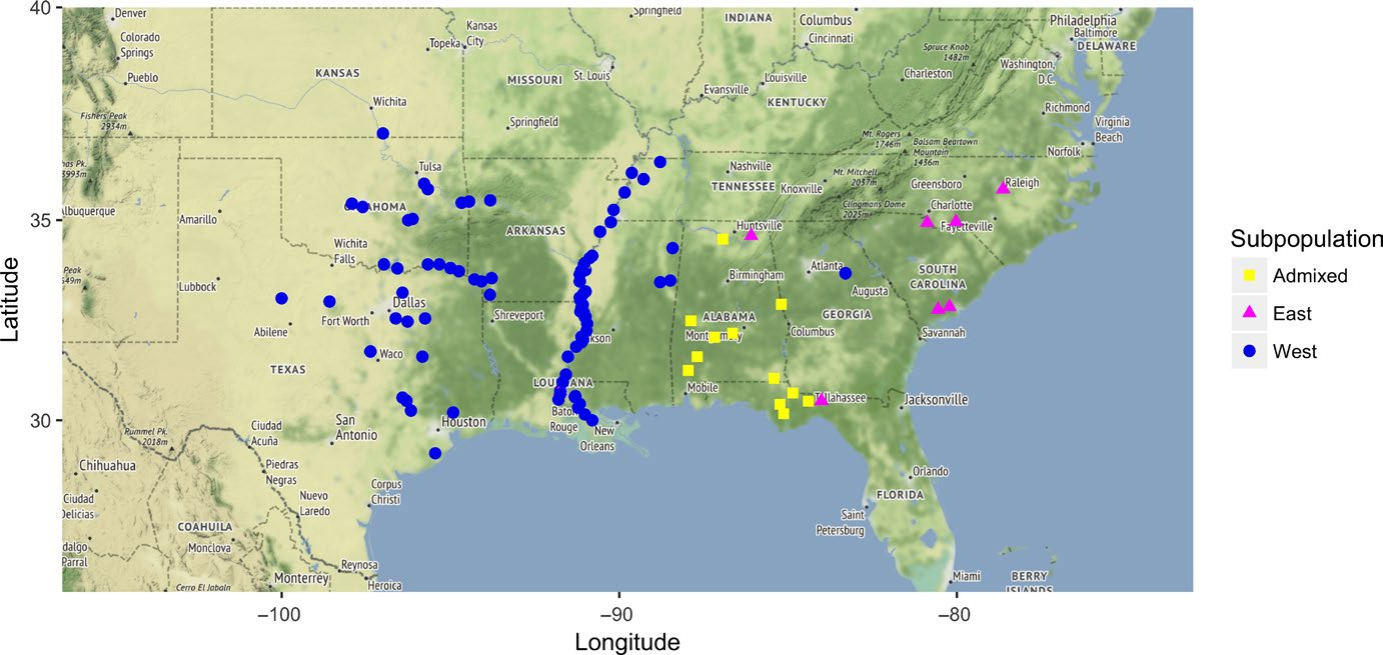
Geographic distribution of subpopulations identified in *Populus deltoides* with STRUCTURE when assuming two groups (*K* = 2)

**Figure 2:**
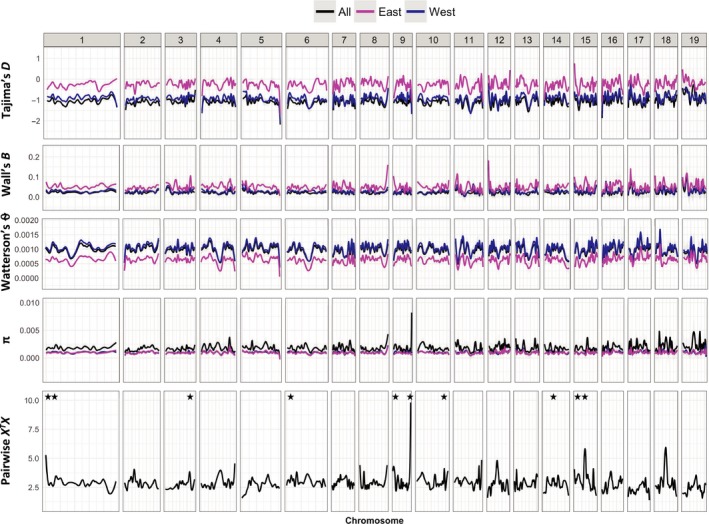
Genomewide distribution of Tajima's *D*, Wall's *B*, nucleotide diversity (θ_W_ and π), and population differentiation (*X*^*T*^*X*) by subpopulation in *Populus deltoides* when assuming two groups (*K* = 2; subpopulation East‐K2: magenta; subpopulation West‐K2: blue; complete population: black). The approximate location of the *X*^*T*^*X* outliers detected is marked with a black star

Identification of loci under selection in natural populations provides insight into the genetic regulation of fitness and adaptation (Kardos et al., 2015). One widely used method to detect selection signatures is the Tajima's *D* statistic (Tajima, 1989), calculated based on the comparison between the number of segregating sites, in the form of θ_W_, and nucleotide diversity, π. Tajima's *D* was calculated by gene with PopGenome (Pfeifer et al., 2014) in each subpopulation using the 555,673 filtered SNPs. Mean Tajima's *D* was positive for the Center subpopulation (0.1354), and negative in all other subpopulations (East: −0.2166; West: −0.42330; West‐MR: −0.7326; Fig. S2, Table S4). Genomewide positive Tajima's *D* may be detected when there is bias toward common alleles (Ramírez‐Soriano & Nielsen, 2009). The latter seems to be the case for the Center subpopulation, which contains less than half the fraction of low‐frequency SNPs (31%) than the total population (77%; Table S5). This can be explained by this subpopulation's small sample size (*N* = 26)—lower frequency polymorphisms likely present in this subpopulation would require a larger sample to be detected, and their frequency estimated. Thus, further analysis for detection of signatures of selection and population differentiation was performed on subpopulations detected with STRUCTURE when assuming two subpopulations (Figure 1, Table S2), where samples were separated into a western (West‐K2) and an eastern (East‐K2) group. This reflects the first level of hierarchical structure present in the core *P. deltoides* population (Puechmaille, 2016). When using these two groups, all samples from the Center subpopulation were excluded for being classified as admixed, and the West and West‐MR subpopulations were merged into one. Using an ancestry coefficient cutoff of 0.8, 172, and 188 samples were assigned to the West‐K2 and East‐K2 subpopulations, respectively, with a pairwise *F*_ST_ value of 0.072. Heterozygosity, nucleotide diversity, Tajima's *D*, and effective population size for these two groups are reported in Table S4. Both subpopulations showed negative mean Tajima's *D* values (Figure 2, Table S4) and a high proportion of low‐frequency SNPs (Table S5). The West‐K2 subpopulation showed higher θ_W_ than East‐K2 along all chromosomes, while π was very similar in both subpopulations (Figure 2).

Tajima's *D* outlier loci were identified in the East‐K2 and West‐K2 subpopulations selecting the genes with the 1% top negative values from the Tajima's *D* distribution obtained for all genes analyzed. These genes are more likely to be affected by selective sweeps or purifying selection. This gene set was enriched for the GO annotation protein binding; PFAM annotations zinc finger, SET, and TIFY domains; KEGG annotation EREBP‐like transcription factor; and for genes targeted by the homeodomain transcription factors ARBORKNOX1 (ARK1) and ARK2 (Table S6). Of the 168 outlier loci selected from each subpopulation, 25 loci were detected in both groups (Table S7), indicating that they may play an important functional role in the species as a whole. Although outlier loci with positive Tajima's *D* can be under diversifying selection, these have to be interpreted cautiously and were not analyzed further because they can also arise from duplicated regions in the genome, causing differences between regions to be mistaken for polymorphism inside a gene.

Linkage disequilibrium among segregating sites can also be used to detect departures from neutrality and has been incorporated in the calculation of Wall's *B* statistic (Wall, 1999). This statistic was calculated by gene with PopGenome (Pfeifer et al., 2014) in the total population and the East‐K2 and West‐K2 subpopulations using the 555,673 filtered SNPs. Distribution of Wall's *B* along chromosomes (Figure 2) shows that LD is higher in the East‐K2 subpopulation than in West‐K2 or the total population. This finding was confirmed when analyzing LD decay with distance within genes, with LD decaying faster in the total population and in the West‐K2 subpopulation, compared to East‐K2 (Fig. S3). The allele frequency threshold used to select SNPs for this analysis greatly influenced the estimated LD decay. When including low‐frequency SNPs (excluding only SNPs with MAF <0.01), LD decayed below a threshold of 0.2 after ~60 bp in all populations (total population, East‐K2 and West‐K2). Analysis of common SNPs revealed that LD decayed below a threshold of 0.2 after 1,472 bp when using SNPs with MAF >0.05; and after 5,118 bp when using a MAF cutoff of 0.1 in the total population. A similar LD decay pattern was observed for the West‐K2 subpopulation, while in the East‐K2 subpopulation LD decayed below a threshold of 0.2 at approximately 1.6 to two times greater distances than in West‐K2 (Table S8). These values are an average for the ~16,500 genes analyzed across the 19 chromosomes. An LD decay of ~5 kb (*r* ^2^ threshold of 0.2) was observed in the total population, when using common SNPs (MAF > 0.1). This estimate is largely in agreement with the distance reported previously for *P. trichocarpa*, when the same MAF threshold was used (Slavov et al., 2012).

### 3.4 Genomewide patterns of subpopulation differentiation in *Populus deltoides*

Loci under divergent selection involved in local adaptation can be detected through *F*_ST_ outlier tests, designed to identify genomic locations that show large allele frequency differences between populations (Lotterhos & Whitlock, 2014). Genomewide population differentiation was assessed here with the *X* ^*T*^ *X* statistic implemented in BAYENV2 (Günther & Coop, 2013). This statistic is similar to *F*_ST_, but it was chosen over the latter because it allows correcting for common evolutionary history between subpopulations. This is achieved through the use of a variance–covariance matrix of allele frequencies among populations, estimated with putatively neutral loci. In this study, 1,800 intergenic SNPs were used to estimate the covariance matrix. Also, to avoid the detection of large numbers of false positives, the same set of intergenic SNPs was used to obtain the null *X* ^*T*^ *X* distribution (Fig. S4). *X* ^*T*^ *X* was calculated for 223,643 genic filtered SNPs with MAF ≥ 0.1. Empirical *p*‐values were calculated for the genic SNPs, and an FDR significance level of 5% was used to declare significant genic *X* ^*T*^ *X* outliers (Fig. S4). A total of 17 genes (33 SNPs) were identified as having significantly different allele frequencies in the East‐K2 and West‐K2 subpopulations (Fig. S5, Table S9). The genomic location of some significant outliers can be seen as a peak in the distribution of the *X* ^*T*^ *X* statistic along chromosomes (Figure 2). Genetic diversity (π) was lower for most outlier genes (15/17) when compared to the mean diversity for all genes analyzed in the two subpopulations. A test for gene function enrichment revealed that aspartyl proteases and genes targeted by the homeobox transcription factors ARK1, ARK2, and popCORONA (PCN) were overrepresented in this gene set (Table S6).

### 3.5 Environmental association analysis

One common goal of population genomics studies in natural populations is the identification of functionally relevant genes that contribute to the population's adaptation and fitness. Identification of selection signatures in a population (e.g., with Tajima's *D*) and loci under divergent selection between populations (e.g., *X* ^*T*^ *X* outlier loci) are indirect ways of analyzing the genetic component of adaptation. A different strategy is to directly search for associations between markers and environmental variables to identify genes involved in adaptation (Rellstab, Gugerli, Eckert, Hancock, & Holderegger, 2015). Here, an EAA was performed using 168 unrelated *P. deltoides* individuals with known sampling location. Environmental variables were obtained from the WorldClim database. Due to a high correlation between many of the variables, PCA was used to identify those that explained the highest proportion of the environmental variance. Temperature and precipitation variables were analyzed separately, and the first four PCs for these two variable types were found to explain more than 90% of the environmental variance in the sample (Table S10). Next, the individual variables with a higher contribution to the first four PCs were identified, resulting in the selection of six temperature and five precipitation variables for the EAA (Fig. S6, Tables S10 and S11). A latent factor mixed model implemented in the LFMM program (Frichot et al., 2013) was used to identify associations between SNPs and the selected environmental variables. This analysis used 114,261 common SNPs (MAF > 0.05) located in 16,804 genes. In this model, two latent factors were included to simultaneously correct for confounding effects (such as population structure) while testing for marker–environment associations. A total of 2,384 SNPs in 2,033 genes were associated with the variables tested after applying a stringent Bonferroni correction for multiple testing (adjusted alpha value = 0.01/114,261 SNPs = 8.75 × 10^−8^; Fig. S7, Tables S12 and S13). This gene set was enriched for KEGG annotations malate dehydrogenase, proteasome activator subunit 4, and large subunit ribosomal protein L9e, as well as for targets of the homeobox transcription factors ARK1, ARK2 and popBELLRINGER (BLR; Table S6).

**Figure 3:**
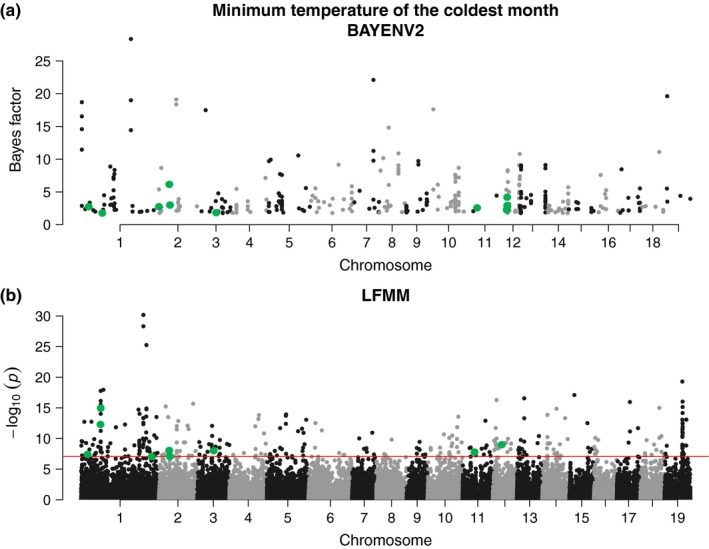
Environmental association analysis with minimum temperature of the coldest month (Bio6) in *Populus deltoides*. (a) Bayes factor obtained with BAYENV2 for the single nucleotide polymorphisms (SNPs) strongly associated with the environmental variable (SNPs ranked in the top 1% Bayes factor and rho). (b) Manhattan plot of the association results obtained with LFMM. The red line indicates a Bonferroni significance level of 0.01. In (a) and (b), SNPs present in genes identified by both methods (BAYENV2 and LFMM) as associated with minimum temperature of the coldest month are shown in green

**Figure 4:**
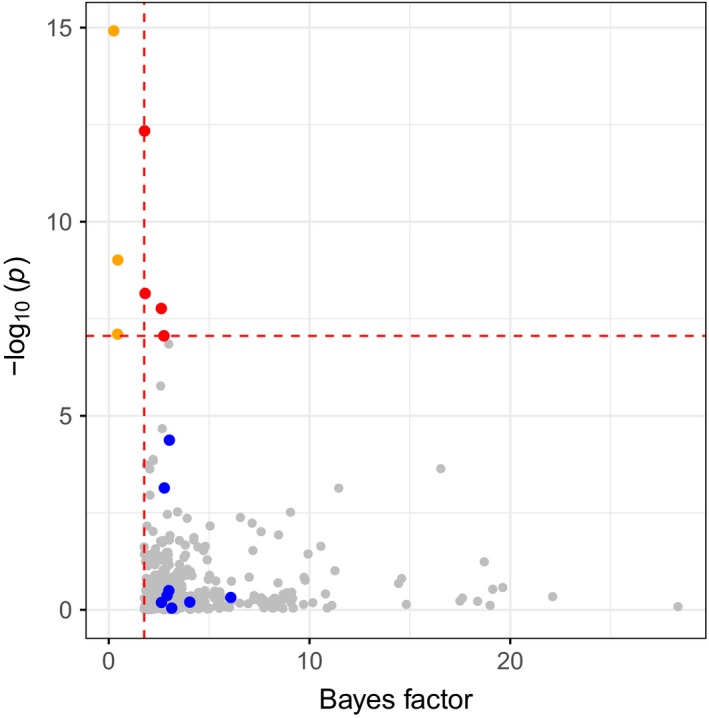
Overlap between single nucleotide polymorphisms (SNPs) associated with the variable “minimum temperature of the coldest month” identified with two different methods (BAYENV2 and LFMM) in *Populus deltoides*. The scatterplot shows the SNPs identified as strong candidates for adaptation by BAYENV2 (those ranked among the 1% highest Bayes factor and 1% highest absolute value of ρ), correlating the Bayes factor obtained with BAYENV2 (*x*‐axis) with the −log_10_ of the *p*‐value obtained with LFMM (*y*‐axis). Four SNPs in four different genes were identified as significant with both methods (red points). Four additional overlapping candidate genes were identified through environmental association with different SNPs among methods. The eight SNPs identifying these additional genes when using BAYENV2 are shown in blue. These four additional genes were identified through five significant SNPs when using LFMM. Two of the SNPs were not included in the analysis with BAYENV2 and are not included in the figure for lack of a corresponding Bayes factor (their *p*‐values were 4.68 × 10^−08^ and 1.97 × 10^−08^). The other three SNPs are shown in orange. All remaining SNPs (gray points) were only significant according to BAYENV2 and not LFMM. The horizontal dashed line indicates the 1% significance threshold after Bonferroni correction for multiple testing applied to the LFMM results (*p*‐value = 8.75 × 10^−08^), and the vertical dashed line indicates the Bayes factor cutoff used to select the top associations (Bayes factor = 1.766)

For comparison, an EAA was also carried out using BAYENV2. In contrast to LFMM, which does not require population information, BAYENV2 searches for the association between the allele frequencies found in a population and a given environmental variable. For this reason, the 168 trees with known sampling location were grouped into 50 populations according to latitude, longitude, and altitude (Table S1). Mean population values were obtained for each environmental variable and standardized prior to the association analysis (Table S14). Out of the markers selected for the EAA with LFMM, a set of 79,969 SNPs with less than 25% missing data was analyzed with BAYENV2. A set of 1,876 genes (2,522 SNPs) associated with one or more environmental variables was discovered (Fig. S8, Tables S12 and S15). Many functional categories were overrepresented in this gene set, including PFAM annotations WD domain/G‐beta repeat, NB‐ARC domain, and leucine‐rich repeat; KEGG annotation ethylene receptor; and genes targeted by the homeobox transcription factors ARK1, ARK2, and BLR (Table S6). Thirty‐five of the gene–environment associations discovered by BAYENV2 were also identified in the EAA carried out with LFMM (Table S16). The environmental variable showing the greatest overlap between methods (eight genes) was minimum temperature of the coldest month (Figures 3 and 4). In addition, 115 genes were correlated with the environment by both methods, but the environmental variable showing the association was different between them (Table S17).

## 4 DISCUSSION

Forests around the globe are facing great challenges because of climate change. Their fate will depend on the capacity of tree species to migrate or adapt. Our ability to predict their future and possibly assist in their migration depends on a detailed knowledge of the factors that govern their responses to the environment (Aitken et al., 2008). A better understanding of the genetic regulation of adaptation is fundamental for the development of adequate management and conservation strategies under a changing climate (Porth et al., 2015). In recent years, population genomics has been used to study tree populations, identifying genes under selection involved in local adaptation (Evans et al., 2014; Geraldes et al., 2014; Holliday et al., 2016; Zhou et al., 2014). The great advantage of population genomics studies over smaller‐scale population genetics studies is that the former allow identifying and correcting for genomewide demographic effects, increasing the power to detect locus‐specific effects (Stinchcombe & Hoekstra, 2008). Here, we present the first population genomics study assessing population structure, genetic diversity, LD, population differentiation, and adaptation in *P. deltoides*, a species lacking this information at a genomewide scale.

A population structure analysis conducted previously (Fahrenkrog et al., 2017) on the *P. deltoides* population used in this study revealed the presence of four subpopulations, following a longitudinal gradient from east to west and showing evidence of hierarchical structure. This population structure pattern agrees with the landscape over which *P. deltoides* is naturally distributed and is consistent with a scenario of isolation by distance. In *P. trichocarpa*, isolation by distance was identified as a main driver of population structure (Geraldes et al., 2014; Zhou et al., 2014). The eastern and western subpopulations are separated by a known phylogeographic barrier, the Appalachian Mountain discontinuity (Soltis, Morris, McLachlan, Manos, & Soltis, 2006). Although the presence of population structure was evident, the differentiation between subpopulations was weak (*F*_ST_ between 0.022 and 0.106) as expected for outcrossing forest tree species distributed over extensive geographic regions and with long‐distance pollen and seed dispersal capacity (Ingvarsson, 2010; Slavov & Zhelev, 2010). Low population differentiation could also be the consequence of recent divergence among populations (Holsinger & Weir, 2009), an aspect not analyzed in the present study.

Based on exome resequencing data, we assessed the genetic diversity and effective population size in *P. deltoides*. Mean diversity obtained by gene (θ_W_ = 0.00100, π = 0.00170) was found to be similar to previous reports for a small number of genes in *P. deltoides* (Breen et al., 2009). Consistent with purifying selection acting on coding regions, mean intergenic diversity (θ_W_ = 0.00494) was greater than genic diversity and both estimates were slightly lower than values reported for *P. trichocarpa* (Zhou et al., 2014). An effective population size of ~6,700 was estimated for the total population under study, based on diversity calculated by gene. This value is also similar to the effective population size reported for *P. trichocarpa* (Slavov et al., 2012; Zhou et al., 2014). Effective population size estimated considering intergenic diversity (~32,900) was five times larger than the one based on genes. In the latter, SNPs were identified only in the regions targeted by probes designed for sequence capture, corresponding mainly to exons. Polymorphisms in introns were largely not assessed in this study, biasing the genic diversity estimates downwards. Additionally, the filters applied to select high‐confidence SNPs, especially the removal of markers based on call rate, most likely excluded true variants from further analysis, affecting both (genic and intergenic) *N*_e_ estimates. Genic *N*_e_ should be taken as a lower bound for this parameter, which is more likely closer to the intergenic estimate. This result highlights the importance of carefully choosing the markers to be used in the estimation of *N*_e_, parameter that varies greatly across different genomic features (Ellegren & Galtier, 2016). The high levels of genetic diversity and large *N*_e_ identified here in *P. deltoides* indicate that this species has good potential to adapt to new environmental conditions arising under a changing climate (Aitken et al., 2008). Also, the high levels of genetic diversity present in this species are an excellent source of new alleles that can be incorporated into breeding programs for poplar improvement (Vanholme et al., 2013).

Conflicting results have been reported for the extent of LD in *P. trichocarpa*, with genomewide studies reporting LD to decay below a threshold of 0.2 at a distance of 5–7.5 kb (Slavov et al., 2012; Zhou et al., 2014), and a study based on a reduced number of candidate genes reporting LD to decay below the same threshold after only 200 bp (Wegrzyn et al., 2010). In the study reported here, LD decay was assessed with different SNP sets selected based on MAF thresholds. When including low‐frequency SNPs in the analysis together with common variants, LD decayed below a threshold of 0.2 after 63 bp in the total population. Removal of SNPs with MAF <0.05 revealed an LD decay distance of ~1.5 kb, while removal of SNPs with MAF <0.10 increased the LD decay distance to ~5 kb. The latter value falls in the same range reported for *P. trichocarpa* when genomewide LD was assessed, because those studies also applied allele frequency filters keeping only common SNPs (MAF ≥ 0.1). Contrastingly, the candidate gene study reported previously for *P. trichocarpa* (Wegrzyn et al., 2010) did not filter SNPs by allele frequency, and faster LD decay was detected. The LD extent observed in *P. deltoides* is advantageous for the successful implementation of genomewide association studies (GWAS) in this species, methodology that relies on LD between causative and tested markers to identify genomic regions associated with a trait or variable of interest (Platt, Vilhjálmsson, & Nordborg, 2010). LD levels observed in this population when using common markers are sufficiently high for GWAS to be successful (e.g., Fahrenkrog et al., 2017), but also low enough to achieve high resolution (Lu et al., 2011). In this population, linkage mapping is thus likely to be more effective for common markers, variants that most GWAS methods have also higher power to detect. Additionally, the increase in LD expected around loci under positive selection (Nielsen, 2005) is an advantage when searching for associations with adaptive traits, further increasing the power of GWAS.

Evolutionary forces such as selection shape the genetic variation present in current populations (Oleksyk, Smith, & O'Brien, 2010). Thus, analysis of genetic polymorphism across the genome can reveal regions under selection. We applied three different methods to identify these regions, which are likely to contain genes involved in local adaptation. First, Tajima's *D* statistic, which summarizes the allele frequency spectrum and identifies loci where allele frequencies deviate from the expectation, was obtained to identify genes under selection. Mean Tajima's *D* was negative for the complete population and for most subpopulations analyzed. With the exception of the Center subpopulation, where positive values were probably due to small sample size and inadequate sampling of low‐frequency alleles, negative values for genic regions in all other populations indicate the presence of a larger number of rare alleles than expected under the standard neutral model (Nielsen, 2005). This excess of rare alleles can be the consequence of purifying selection keeping deleterious mutations at low frequency in the genic regions analyzed. It can also be the consequence of demographic changes influencing diversity in the entire genome, like population growth after an ancient bottleneck (Holliday, Yuen, Ritland, & Aitken, 2010). Population size reduction during the last glacial period and population growth after this bottleneck has shaped demography in other tree species (Heuertz et al., 2006; Holliday et al., 2010; Ingvarsson, 2008; Pyhäjärvi et al., 2007; Zhou et al., 2014) and is also a plausible scenario for *P. deltoides*, but demography has not been assessed in this species to date. In the absence of a demographic study in *P. deltoides* and because demography can confound signatures of selection causing false‐positive Tajima's *D* outliers, this statistic was used to complement the information obtained with other tests.

Second, to identify loci under divergent selection among subpopulations, population differentiation was assessed with the *X* ^*T*^ *X* statistic implemented in BAYENV2. *X* ^*T*^ *X* has advantages over the more commonly used *F*_ST_ statistic for this purpose, because it corrects for common evolutionary history (Günther & Coop, 2013), making it more powerful to detect local adaptation (Lotterhos & Whitlock, 2014). Additionally, the calculation of empirical *p*‐values further reduces the number of false‐positive loci detected (Lotterhos & Whitlock, 2014). This procedure led to the discovery of 17 genes with large allele frequency differences between the East‐K2 and West‐K2 subpopulations (Table S9). Most *X* ^*T*^ *X* outlier SNPs in genes within the same chromosome showed high levels of LD, resulting in 11 genomic regions diverging between populations. The differentiation among populations observed at these loci and decrease in nucleotide diversity observed in most of them could be due to divergent selection and local adaptation, but it could also be caused by other processes. For example, low genetic diversity within populations not related to adaptation could be causing these outliers (Cruickshank & Hahn, 2014; Vijay et al., 2017). Other processes that can cause spurious population differentiation outliers are background selection (selection against deleterious alleles; Charlesworth, Morgan, & Charlesworth, 1993), specieswide selective sweeps leading to transient outlier loci, cryptic hybrid zones, and stochastic effects in expanding populations (Bierne, Roze, & Welch, 2013; Savolainen, Lascoux, & Merilä, 2013; Vijay et al., 2017). Further characterization of the *X* ^*T*^ *X* outlier loci and elucidation of their biological function will help determine whether they play a role in adaptation and harbor useful alleles to be incorporated into breeding programs targeted to specific geographic regions. They might also be informative to select material for conservation or assisted migration efforts in the face of climate change.

Finally, an EAA was conducted to identify genes associated with 11 bioclimatic variables that explained a high proportion of the environmental variance between samples. Two methods (LFMM and BAYENV2) designed to detect environmental associations including correction for population structure were chosen for this analysis. Despite this correction, LFMM detected a large number of associations (2,033, Table S13) at a 1% significance level after a Bonferroni correction for multiple testing. The analysis with BAYENV2 also identified a large number of loci (1,876, Table S15) associated with the environment, with 35 of them overlapping with the significant genes discovered with LFMM (Table S16). Within most overlapping genes, each method identified different SNPs as associated with a trait. Also, the overlapping genes did generally not contain the most significant associations from either method (see example for minimum temperature of the coldest month in Figures 3 and 4). In general, the limited overlap observed between EAA methods could be due to the smaller SNP set analyzed with BAYENV2 (LFMM: 114,261 SNPs in 16,804 genes; BAYENV2: 72,969 SNPs in 15,204 genes). Analysis of less genes and also less SNPs by gene with BAYENV2 likely caused a reduction in the probability of identifying associations. The two EAA methods combined provided an extensive list of candidate genes, suggesting that adaptation to climate is of complex genetic nature in *P. deltoides*, as is often the case in plants (Savolainen et al., 2013).

**Figure 5:**
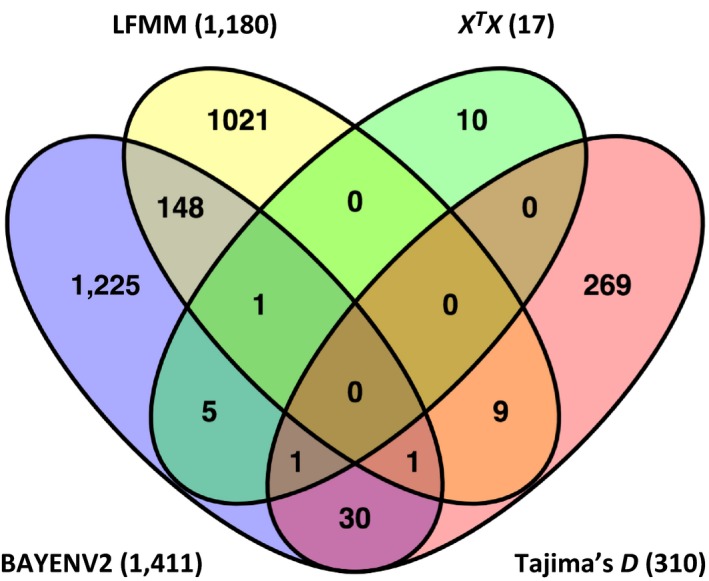
Overlap between methods used to identify genes under selection in *Populus deltoides*. The total number of genes identified is noted in parentheses next to the name of the method. The number of genes is lower than the number of associations reported in the text for LFMM, BAYENV2 and Tajima's *D*, because some genes were associated with more than one variable in the environmental association analysis with LFMM and Bayenv2 and some genes were Tajima's *D* outliers in the two populations analyzed

When comparing the results obtained with the four methods used to detect loci under selection, Tajima's *D*,* X* ^*T*^ *X*, and EAA with LFMM and BAYENV2, one gene stands out for being identified by three of them: Potri.006G021900 (POPTR_0006s02270) (Figure 5, Table S18). This *X* ^*T*^ *X* outlier also showed a very low Tajima's *D* value in the East‐K2 subpopulation and was identified by BAYENV2 as associated with mean temperature of the driest quarter. This gene encodes a putative topoisomerase II‐associated protein orthologous to PAT1 in *Arabidopsis thaliana* (AT1G79090), an mRNA decay factor involved in post‐transcriptional gene regulation and innate immune response to biotrophic and necrotrophic pathogens (Roux et al., 2015). In a genomewide association study, we recently conducted on the same *P. deltoides* population analyzed here (Fahrenkrog et al., 2017), a SNP present in this gene was positively associated with wood lignin content. Lignin is a polymer that increases rigidity and resistance of plant cell walls, playing an important role in the plant's defense against pathogens (Bhuiyan, Selvaraj, Wei, & King, 2009). This indicates that gene Potri.006G021900 could be involved in pathogen response through regulation of lignin biosynthesis.

Other interesting candidates for adaptation are genes identified by two of the three methods used to identify genes under selection (Table S18). This is the case for another *X* ^*T*^ *X* outlier, Potri.015G065400 (POPTR_0015s07640), correlated with environmental variables in the EAA (precipitation in the month of May with BAYENV2 and maximum temperature in the warmest month with LFMM). This gene's ortholog in *A. thaliana* (AT3G01470) encodes a homeodomain leucine zipper class I (HD‐Zip I) transcriptional activator involved in leaf and hypocotyl development. It is also involved in the plant's response to blue light. According to the PopGenIE database (Sjödin et al., 2009), the expression profile in different poplar tissues shows this gene to be downregulated in dormant and prechilling buds and in dormant and expanding flowers. It is also upregulated in leaves in response to drought, expression pattern that might explain the correlation between this gene and the two climate variables mentioned above.

Five additional *X* ^*T*^ *X* outliers were also correlated with the environment after analysis with BAYENV2 (Table S18). Among those genes, the SNPs that identified two of them as *X* ^*T*^ *X* outliers are predicted by SNPEFF (Cingolani et al., 2012) to be missense mutations, more likely to affect protein function. The first gene, Potri.003G178800 (POPTR_0003s17720), was associated with maximum temperature in the warmest month and has been found to be upregulated in dormant bud, young‐expanding leaves, dormant flowers and wood, and downregulated in leaves under drought conditions in poplar trees. It encodes a putative mitochondrial/chloroplast ribosomal protein S15 and is orthologous to gene AT1G80620 in *A. thaliana*. The second gene, Potri.009G027300 (POPTR_0009s03240), is orthologous to gene AT3G46130 in *A. thaliana*, which encodes a putative MYB transcription factor involved in flavonol biosynthesis. In poplars, this gene is upregulated in roots and downregulated in buds (dormant and prechilling), young‐expanding leaves, and dormant and expanding flowers. In this study, this gene was correlated with mean diurnal temperature range. The overlap between genes associated with climate and Tajima's *D* outliers provided an additional list of 40 candidate genes for adaptation (LFMM/BAYENV2/Tajima's *D*: 1 gene; BAYENV2/Tajima's *D*: 30 genes; LFMM/Tajima's *D*: nine genes; Table S18). These genes are also interesting targets to be functionally characterized in *P. deltoides* to verify their suggestive role in adaptation.

To gain insight into the biological function of the set of candidate genes identified, we carried out a functional enrichment analysis. The complete list of candidate genes (including Tajima's *D* and *X* ^*T*^ *X* outliers, as well as genes associated with the environment) was enriched for genes involved in ubiquitin‐dependent protein catabolism (biological process GO:0006511), protein binding (molecular function GO:0005515), nucleoside‐triphosphatase activity (GO:0017111), pyrophosphatase activity (GO:0016462), protein–protein interactions (WD domain, G‐beta repeat, PF00400), secondary metabolism (Cytochrome P450, PF00067), and ethylene receptors (K14509), among others (Table S6). It was also enriched for targets of transcription factors involved in secondary growth and wood development (ARK1, ARK2, BLR, and PCN). When analyzing the candidate genes identified by each method separately, they were enriched for different functions. Tajima's *D* outliers were enriched for transcription factors; *X* ^*T*^ *X* outliers were enriched for genes involved in protein degradation; LFMM candidate genes were enriched for the enzyme malate dehydrogenase (involved in pyruvate metabolism and carbon fixation; Edwards & Andreo, 1992) and proteasome activator subunit 4 (involved in degradation of histones during DNA damage response, Book et al., 2010); and BAYENV2 candidate genes were enriched for genes involved in disease resistance, protein degradation, ethylene signaling, RNA splicing, protein synthesis, and stress response, among others (Table S6). The only category shared by all candidate gene sets is the enrichment for targets of the transcriptional regulators ARK1 and ARK2 involved in wood formation. All enriched functional categories are essential for plant growth, development, and interaction with the environment, and lend further support to the hypothesis that the genes identified in this study are involved in adaptation.

Although the combination of methods applied here resulted in a list of interesting candidate genes, the somewhat reduced overlap between methods could be due to the use of different datasets for each analysis. Tajima's *D* was assessed by gene in the East‐K2 (*N* = 188) and West‐K2 (*N* = 172) subpopulations using all filtered SNPs, *X* ^*T*^ *X* was estimated between both subpopulations using SNPs with MAF ≥0.1, and the EAA was conducted on 168 individuals with known sampling location using consensus SNPs with MAF ≥0.05 without call rate filter for analysis with LFMM and excluding SNPs with more than 25% missing data for analysis with BAYENV2 (Fig. S1). The analysis that detected the lowest number of genes putatively under selection was the *X* ^*T*^ *X* outlier test, which might be a result of the low false‐positive rate reported for this method. It might also be caused by the calculation of empirical *p*‐values based on the *X* ^*T*^ *X* distribution for intergenic SNPs, which would decrease the power to detect true associations if the intergenic SNPs are not selectively neutral as assumed (Lotterhos & Whitlock, 2014). Another reason for the small number of outliers detected could be that the two populations analyzed are distributed over a wide geographic range, decreasing the environmental resolution of the analysis. On the other hand, the EAA conducted with LFMM identified the highest number of putatively selected loci, and many could be false positives. This might be the consequence of insufficient correction for population structure, confounding factor that is known to generate spurious associations in this kind of analysis (Rellstab et al., 2015). In spite of the concerns mentioned above, limited agreement between different methods is expected (Lotterhos & Whitlock, 2015). For example, a population genomics study based on whole‐genome resequencing of a natural *P. trichocarpa* population that analyzed a large number of polymorphisms and compared results between five different selection scans, found very little overlap between tests (Evans et al., 2014). This limited overlap can be explained by the ability of different methods to identify the effects of different models of selection, with outlier tests more suited to identify hard selective sweeps and EAA capable of detecting small changes in allele frequency due to selection acting on standing genetic variation (Evans et al., 2014; Sork et al., 2013). Similarly, in the present study different methods for the identification of regions under selection provided their own interesting list of candidate genes for adaptation and fitness in *P. deltoides* to be analyzed in more detail in the future.

Interestingly, when comparing the candidate genes for adaptation identified in the *P. trichocarpa* population genomics study based on five different selection scans (Evans et al., 2014) with the candidate genes identified here in *P. deltoides*, a set of 360 genes is shared between both studies (Table S19). This corresponds to 2% of all genes analyzed and to 13.2% of all selection candidates in *P. deltoides*. The population genomics study in *P. trichocarpa* also assessed the association of genes within candidate selection regions with three adaptive traits (bud flush, bud set, and height; Evans et al., 2014). Among the 360 candidate genes shared between studies, 129 genes were also associated with an adaptive trait in *P. trichocarpa*. Also, 31 of the 360 overlapping genes were identified by two different selection scans in *P. trichocarpa* and one in *P. deltoides*; and 29 genes were identified by two methods in *P. deltoides* and one in *P. trichocarpa*. In addition, one gene was identified by one method in *P. deltoides* and four methods in *P. trichocarpa*. This gene (Potri.007G032700, POPTR_0007s12170), a Tajima's *D* outlier in the West‐K2 subpopulation, also was identified as a selection candidate in *P. trichocarpa* by four scans performed in that species. This gene is orthologous to the gene AT4G36870 that encodes the BEL1‐LIKE HOMEODOMAIN 2 (BLH2) transcription factor also known as SAWTOOTH 1 (SAW1), a negative regulator of growth involved in leaf margin development in *A. thaliana* (Kumar et al., 2007). Finally, three genes were identified by two selection scans in each species. The first gene, Potri.003G139400 (POPTR_0003s13920), showed correlation with four environmental variables in *P. deltoides* (BAYENV2: annual mean temperature, mean diurnal range, and minimum temperature of the coldest month; BAYENV2 and LFMM: mean temperature of the driest quarter). This gene encodes a protein of unknown function, with the product of its ortholog in *A. thaliana* (AT1G64385) predicted to be an integral membrane protein located in the endomembrane system. The second gene, Potri.008G220800 (POPTR_0008s22360), was associated with mean diurnal range (BAYENV2) and was also identified as a Tajima's *D* outlier in the West‐K2 subpopulation. In *P. trichocarpa*, this gene was significantly associated with the adaptive trait bud flush (Evans et al., 2014). Its ortholog in *A. thaliana*, AT3G06880, is annotated as a Transducin/WD40 repeatlike superfamily protein located in the chloroplast and expressed in guard cells. In general, transducin‐like proteins and proteins containing WD40 repeats act in signal transduction and protein–protein interactions (Stirnimann, Petsalaki, Russell, & Mu, 2010). The third gene, Potri.018G093600 (POPTR_0018s10140), showed association with precipitation seasonality (BAYENV2) and precipitation in the warmest quarter (LFMM) in *P. deltoides*, and it was also significantly associated with height in *P. trichocarpa* (Evans et al., 2014). Its ortholog in *A. thaliana* (AT5G57480) encodes a putative AAA+‐type ATPase expressed during flowering in the petal differentiation and expansion stage.

Although a direct comparison between this study and the population genomics study conducted in *P. trichocarpa* (Evans et al., 2014) is difficult because of the different methods used in both, convergent evolution is a plausible explanation for the identification of a common set of candidate genes for adaptation in both species. The occurrence of convergent evolution was recently reported in conifers, where two distantly related species shared between ten and 18% of locally adapted genes (Yeaman et al., 2016). These findings indicate that sometimes adaptation to environmental factors occurs through mutations in the same genes in different species. Alternative explanations to the identification of adaptive genes common to both species are shared standing genetic variation and hybridization. These species diverged relatively recently and natural hybridization occurs in the northwestern part of *P. deltoides*’ range (Eckenwalder, 1996). A comparative study of local adaptation in different poplar species is needed to elucidate the origin of these findings.

In a GWAS conducted previously on the same *P. deltoides* population analyzed here (Fahrenkrog et al., 2017), we found that purifying selection seemed to be keeping polymorphisms with a negative correlation with wood lignin content at low frequencies in the population. Here, we analyzed the overlap between the set of 307 genes significantly associated with a trait in the GWAS study and the candidate genes for adaptation identified in this study. An overlap of 57 genes was found, with 54 genes being associated with wood lignin content. This finding provides new evidence for wood lignin content being a trait under selection and likely involved in adaptation to the environment.

In summary, the population genomics study reported here for *P. deltoides* found evidence of population structure in the sample analyzed, but with low differentiation between subpopulations. Overall genetic diversity was found to be high and LD low, with the subpopulation identified eastern of the Appalachian Mountains being less diverse than the subpopulations identified western of that phylogeographic barrier. Genomewide scans for selection and environmental association analyses identified genes putatively involved in local adaptation, providing good candidates for validation in follow‐up studies. Interestingly, many adaptive genes were also identified as such in a different population genomics study conducted in *P. trichocarpa* (Evans et al., 2014), indicating that these genes might be undergoing convergent evolution. This study constitutes the first assessment of genetic diversity and local adaptation in *P. deltoides* throughout the southern part of its range, information that will be very useful to guide management and breeding strategies for this species in the future.

## DATA ACCESSIBILITY

1. DNA sequences: NCBI SRA accession number SRP066162.
2. Sampling locations: Supporting Information, Table S2.
3. Climate data: Supporting Information, Table S11.

## CONFLICT OF INTEREST

None declared.

## AUTHOR CONTRIBUTIONS

A.M.F. performed the research, data collection, data analysis and interpretation, and wrote the manuscript; L.G.N. performed the research, data collection, data analysis and interpretation; M.F.R.R.Jr. performed data analysis and interpretation; C.D. designed and performed the research and data collection; R.D. and W.B.B. performed data analysis and interpretation; M.K. planned and designed the research, performed data analysis and interpretation, and wrote the manuscript.
