# Supplementary material for: Population genomics of the eastern cottonwood (Populus deltoides)
Source: Ecol Evol. 2017 Oct 10;7(22):9426–40. doi: 10.1002/ece3.3466 (PMC5696417; doi:10.1002/ece3.3466)
Supplement: Supplementary file 1 [file ECE3-7-9426-s001.docx]

**SUPPLEMENTARY FIGURES**

**TITLE: Population genomics of the eastern cottonwood (*Populus deltoides*)**

**AUTHORS:** Annette M. Fahrenkrog^1,2^, Leandro G. Neves^1,2§^, Márcio F. R. Resende Jr. ^1,3§^, Christopher Dervinis^1^, Ruth Davenport^4^, W. Brad Barbazuk^2,4,5^, Matias Kirst^1,2,5^

**AFFILIATIONS:** ^1^School of Forest Resources and Conservation, University of Florida, P.O. Box 110410, Gainesville, FL 32611, USA; ^2^Plant Molecular and Cellular Biology Graduate Program, University of Florida, P.O. Box 110690, Gainesville, FL 32610, USA; ^3^Genetics and Genomics Graduate Program, University of Florida, P.O. Box 103610, Gainesville, FL 32610, USA; ^4^Biology Department, University of Florida, P.O. Box 118525, Gainesville, FL 32611, USA; ^5^University of Florida Genetics Institute, University of Florida, P.O. Box 103610, Gainesville, FL 32611, USA; ^§^Present Address: RAPiD Genomics LLC, 756 2^nd^ Avenue, Gainesville, FL, 32601, USA

**KEYWORDS:** *Populus deltoides*, eastern cottonwood, exome capture, population structure, genetic diversity, local adaptation

**AUTHOR FOR CORRESPONDENCE:**

Matias Kirst

School of Forest Resources and Conservation, University of Florida, P.O. Box 110410, Gainesville, FL 32611, USA; phone: +1 352 846 0900; fax: +1 352 392 1707; email: [mkirst@ufl.edu](mailto:mkirst@ufl.edu)

**RUNNING TITLE:** ***Populus deltoides* population genomics**

**Figure S1:** Workflow of the population genomics study and environmental association analysis conducted on a natural *Populus deltoides* population. Information pertaining to the population genomics study is shown in blue, environmental association analysis (EAA) in orange, and a previous genome-wide association study conducted on the same population (Fahrenkrog *et al.* 2017) is shown in grey. SNPs: single-nucleotide polymorphisms; MAF: minor allele frequency; LD: linkage disequilibrium; PCA: principal component analysis; π: nucleotide diversity; θ_W_: Watterson’s estimator of nucleotide diversity.

**Figure S2:** Genome-wide distribution of Tajima’s D and nucleotide diversity (θ_W_ and π) by subpopulation when assuming four subpopulations in *Populus deltoides*. The approximate location of the centromeres is marked with a black triangle at the bottom of the figure for each chromosome.

**Figure S3:** Linkage disequilibrium decay with distance within genes in chromosome 1 in the total *Populus deltoides* population and by subpopulation using SNPs with different allele frequency cutoffs. LD decay curves and the distance after which LD decays below a threshold of 0.2 (grey dashed line) are shown in red.

** Figure S4:** Intergenic and genic *X^T^X* distributions used to identify outlier loci in *Populus deltoides*. Genic SNPs with *X^T^X* values above the red arrow are significant outliers.

**Figure S5:** *X^T^X* outlier loci allele frequency comparison among the East-K2 and West-K2 subpopulations in *Populus deltoides*. The most frequent allele in one subpopulation is the least frequent in the other.

**Figure S6:** Temperature and precipitation variables selected for the environmental association analysis in *Populus deltoides*. Temperature is measured in °C and precipitation in mm.

**Figure S7:** Manhattan and q-q plots of the genetic associations identified with eleven environmental variables using LFMM in *Populus deltoides*. The red line in the Manhattan plots marks a 1% significance level after Bonferroni correction for multiple testing.

Figure S7: Continued.

Figure S7: Continued.

Figure S7: Continued.

**Figure S8:** Manhattan plots of the genetic associations identified with eleven environmental variables using BAYENV2 in *Populus deltoides*. The plots show Bayes factor (left) and the absolute value of rho (right) for the SNPs ranked in the top 1% of both statistics.

Figure S8: Continued.

Figure S8: Continued.

Figure S8: Continued.
